# Literacy-related factors and knowledge of patient rights charter: evidence from nurses in selected hospitals in Ghana

**DOI:** 10.1186/s12912-024-01739-w

**Published:** 2024-01-22

**Authors:** John Foster Atta-Doku, Gordon Abekah-Nkrumah, Jacqueline Nkrumah, Prince Owusu Adoma

**Affiliations:** 1 Faculty of Health, Allied Sciences and Home Economics Education, Department of Health Administration and Education, University of Education Winneba, Central Region, West Africa, P. O. Box, 25, Winneba, Ghana; 2 Business School, Department of Health Services Management, University of Ghana, Greater Accra Region, West Africa, P. O. Box, 75, Accra-Legon, Ghana

**Keywords:** Comprehension, Patient rights Charter, Nurse, Literacy, Policy

## Abstract

**Background:**

Systems of across the world have developed and implemented patient rights policies to protect and improve the provider-patient relationship. The Patient Charter of Ghana was developed in 2002 to improve service quality and protect patients’ rights. However, it is not yet known whether those at the frontline of healthcare delivery can read and understand the contents of the charter. While studies have explored the socioeconomic and institutional level factors related to awareness and knowledge of the Patient Rights Charter, there is a lack of literature on its readability and comprehensibility among nurses. This study assesses nurses’ knowledge of the Patient Rights Charter and associated literacy-related factors.

**Method:**

An exploratory cross-sectional design and quantitative methods were used to collect data on knowledge, comprehension, and readability of the Patient Rights Charter. 205 nurses from four district hospitals in the Central Region were recruited using proportional and total enumeration sampling. Data were collected using structured questionnaires and were processed using SPSS (version 26) and an online text readability consensus calculator (version 2.0). Descriptive and inferential statistical analyses were performed, and data were presented using simple frequencies, readability statistics, and regression output.

**Results:**

The results show the charter is written at a higher reading grade level; Flesch-Kincaid Grade Level (13.36), Simple Measure of Gobbledygook (11.57), and Coleman-Liau Readability Index (14.2). The average reading grade level score was 14. The Gunning Fox Index (15.40) and the Flesch Reading Ease Score (34%) show the patient charter is difficult to read and will require at least 14 years of education to be able to read. 87.3% of nurses were able to read and comprehend the charter. Very few (8.3%) read at frustration level. Nurses’ actual comprehension of the charter was the only significant predictor of knowledge of the charter.

**Conclusion:**

Comprehension of the patient charter is an important predictor of its knowledge. The results emphasize the need to enhance the readability and comprehensibility of the charter for providers. Hospitals can stimulate nurses’ knowledge of the charter by simplifying the charter’s language and deliberately educating nurses on its content.

**Supplementary Information:**

The online version contains supplementary material available at 10.1186/s12912-024-01739-w.

## Background

Following the Universal Declaration of Human Rights (UDHR, 1948–1998), most member countries have instituted patient rights policies to uphold and promote the provider-patient relationship [1]. The 1992 Constitution of Ghana guarantees the right to healthcare and other social services to all people living in Ghana, accentuating the importance the country attaches to the health and well-being of Ghanaians [2]. In 2002, Ghana’s Ministry of Health enacted the Patient Rights Charter (PRC) to stipulate the standard of care patients expect and can demand as a fundamental human right. The PRC of Ghana spells out the rights and responsibilities of all patients in the care delivery process that health facilities are expected to provide and respect irrespective of a patient’s socio-economic background, age, and creed [3].

The introduction of the charter marks a significant stride towards improving patient care and nurturing a patient-centered approach in healthcare delivery [4]. It provides a basis for patients to demand high-quality care and emphasizes the provision of services aligned with patients’ needs and preferences [3, 5]. In spite of the conspicuous display of patients’ rights and responsibilities in various healthcare facilities across Ghana, several studies have reported low awareness and knowledge of the charter and its contents among both patients and healthcare providers [3, 6–8]. Research in Ghana has examined various aspects of the PRC over the years, including its implementation, public awareness, and understanding of the rights and responsibilities of patients and healthcare providers [6, 7, 9].

Literature also reports patient perspectives and the overall impact of the PRC on healthcare delivery. Yet, patient and provider readability and comprehensibility of PRC are scarce in the literature. Readability is an essential statistic for a text. It expresses the difficulty or ease with which readers are able to read and understand a text [10]. Consequently, evaluating the readability of the charter will hone policymakers’ and providers understanding of the textual difficulty level and suitability of the charter’s text for patients and providers from different backgrounds. It is also critical to appreciate whether the target audiences of the charter are able to receive the message as intended by policymakers. The two indices, which are core elements of literacy, can inform the restructuring of the charters’ text, both at the national and institutional levels, and improve the literacy of the PRC in Ghana.

Henrard et al. [11] mentioned the importance of clinician’s health literacy and suggested the need for its integration into healthcare quality improvement efforts. Clinicians’ literacy plays an essential role in assimilating and interpreting complex healthcare policies and guidelines [12, 13]. Clinicians’ literacy emphasizes the multidimensional nature of health literacy and its significance in facilitating the access, comprehension, evaluation, and application of health-related information such as the PRC of Ghana [14]. Nurses play a crucial role in monitoring and evaluating healthcare services, and their ability to read and understand the patient charter is key in this process [15]. Thus, assessing the readability and comprehensibility of the PRC will contribute to the delivery of quality healthcare in Ghana.

Sadly, limited scholarly attention is given to the charter’s readability, understandability, and how they contribute to healthcare professionals’ knowledge, awareness, and adherence to PRC. Thus, stressing the critical need to explore the literacy-related factors and their effect on knowledge of the charter. Employing a quantitative approach, this study addressed the gap by assessing nurses’ knowledge of the PRC of Ghana and associated literacy-related factors. Identifying the specific literacy challenges encountered by nurses will help develop interventions appropriate for improving knowledge and understanding of the PRC among providers.

## Methods

### Study area

The study was conducted in hospitals of some selected districts in the Central Region of Ghana. The region has a literacy rate of 82.9% which is higher than the national average of 77.8% [16]. High literacy rate, along with proximity, formed the basis for choosing the region for the study. The authors’ motivation was that the high literacy rate may translate into knowledge of the charter. The study was conducted in three (3) of the 22 districts in the region. The Effutu Municipality, Agona West Municipality, and Asikuma Odoben Brakwa District were used. The Effutu Municipal has a population of about 77,700 people and a literacy rate of 80.9% [16]. The Agona West Municipal has a population of about 180,000 people, with a high literacy rate of 85.1% [16]. The Asikuma Odoben Brakwa District has a population of approximately 80,000 people and a literacy rate of 63.2% [16].

### Study institution

In total, the four main hospitals in the three districts were chosen for the study. These hospitals were chosen because they are all first-level referral facilities with high patronage and more professional staff. The Trauma and Specialist Hospital, Winneba, is a specialized secondary referral health facility with a bed capacity of one hundred and fifty-six (156) and a staff strength of one hundred and thirty-one nurses (131). The Winneba Municipal Hospital is a public healthcare facility located at Winneba. It has a one hundred and eighteen (118) bed capacity with a staff strength of one hundred and ninety-eight (198) nurses. The catchment area for the two hospitals is the Efutu municipality and they serve as referral centers within the municipality and surrounding districts. The Agona Swedru Municipal Hospital is a government-owned healthcare facility located in the town of Agona Swedru in the Agona West Municipal District. It has a staff strength of two hundred (200) and a bed capacity of one hundred and forty-eight (148). The hospital serves as the primary healthcare facility for the people living in and around the town of Agona Swedru. Our Lady of Grace Hospital is a Catholic hospital at Breman Asikuma in the Asikuma Odoben Brakwa District. It has a bed capacity of one hundred and four (104) with a staff strength of one hundred and sixty-seven (167) nurses. It acts as a referral center for many facilities.

### Study design and sampling procedure

An exploratory cross-sectional design and quantitative methods were employed for the study. The population for the study was all full-time nurses in the selected hospitals. A sample of 248 nurses was estimated for the study based on a total nursing population of 696, considering a confidence level = 95%, *p* = 50%, and margin of error = 5% which yielded 248 nurses. The estimate was performed using an online sample size calculator. The number of nursing staff selected per hospital was proportional to the nursing staff strength of each hospital. Table 1 presents information on the proportional sampling. The calculation was done using the formula: ${n}_{h}=\frac{\left(n\right){N}_{h}}{N}$ where;

n = total sample size of nurses (248)

Nh = stratum size (staff strength of each hospital)

N = total nursing population (696) [17].

The selection of nurses was done through a two-stage sampling procedure. In the first stage, four district hospitals were randomly picked from a sampling frame of district hospitals within the Central Region. The total number of nursing staff projected for the study was recruited based on willingness to participate and informed consent.

**Table 1:** Proportional Sampling of Nurses

Name of Hospital			Population
Trauma & Specialist	$\frac{\left(248\right)131}{131+200+198+167}$	$\frac{\text{32,488}}{696}$	47
Winneba Municipal	$\frac{\left(248\right)198}{131+200+198+167}$	$\frac{\text{49,104}}{696}$	70
Swedru Government	$\frac{\left(248\right)200}{131+200+198+167}$	$\frac{\text{49,600}}{696}$	71
Our Lady of Grace	$\frac{\left(248\right)167}{131+200+198+167}$	$\frac{\text{41,416}}{696}$	60
**Total**			**248**

Source: Field data, August 2023

Regarding the readability assessment of the PRC, the two hundred and fifty (250) words used to construct the cloze test and the Patient Education Material Assessment Tool (PEMAT) assessment were used for the readability test. (Please see additional file 1)

### Data collection instruments

Author-developed instruments adapted from the PRC were used to assess comprehension and knowledge of the patients’ charter. Details on the Cloze Test, PEMAT, and questionnaire on PRC Knowledge are provided as follows.

### The cloze test

In the medical field, cloze tests have been utilized to evaluate students’ knowledge of medical terminology and comprehension of clinical scenarios. Specifically, the multiple-choice cloze test has become a common method for assessing medical knowledge and comprehension [18]. There are several types of cloze tests, including multiple-choice cloze tests, traditional cloze tests, and modified cloze tests. In multiple-choice cloze tests, respondents are presented with a list of options to choose from to complete the cloze, allowing for a more precise evaluation of comprehension. In this study, nurses’ comprehension of the PRC was assessed by the multiple-choice cloze test. The cloze test comprised three (3) sections: a section on the social and demographic data of respondents, knowledge of the PRC, and the cloze test. The 250 words for the cloze items were developed based on a random sample of elements from the charter including access, information, confidentiality, choice, self-determination, and redress from the PRC. Fifteen (15) multiple-choice cloze items, each with four possible answers (A-D) were created out of the 250 words. The deletion of words from the passage was based on the rational deletion method [19]. Nurses were to fill in the blank spaces provided with the correct option to complete the cloze. (Please see additional file 2).

### The PEMAT

The authors adapted the PEMAT to further assess the nurses’ perceived understanding of the 250-word passage used to develop the cloze test. The PEMAT is a scale for assessing the understandability and actionability of patient educational materials [20], developed by the Agency for Healthcare Research and Quality (AHRQ) and has been widely used in healthcare research to evaluate the clarity and understandability of patient education materials [21]. The understandability subscale of the PEMAT was adopted to assess nurses’ perception of the understandability or otherwise of the 250-word passage in the cloze test. Specific questions selected for the assessment included questions 1–5 and 7–11. However, the authors simplified the text of the individual items to make it clearer and comprehensible to the nurses. The text simplification was done by an English Language expert from the Department of English Language at the University of Education Winneba. (Please see additional file 3).

### Validity of data collection instruments

All the test instruments were validated by an assessment and evaluation and health literacy experts. The test materials were examined to ensure they were appropriate and captured essential aspects of the PRC. Multiple rounds of feedback and discussion from the experts were used to refine the contents of the instruments [22]. The cloze test was pretested among 42 nurses from a mission-based hospital and a private hospital. The questionnaires were checked for reliability using Cronbach alpha (cloze test, α = 0.82).

### Data collection

Data collection was done between June to July 2023. Written approval was obtained from the management of selected hospitals including consent to conduct the study in their hospital. The purpose of the study was explained to the nurses and consent for participation was taken from willing respondents. All instruments were self-administered. Knowledge of the PRC was based on respondents’ recall. It was determined by asking respondents to write at least six rights and responsibilities of patients in spaces provided in the section for knowledge. The knowledge section was completed and collected before the responses on the cloze test were provided. The motive was to prevent the respondents from picking clues from the cloze to write the rights and responsibilities of patients. The cloze test was administered to nurses under the supervision of the first and third authors. This was done to prevent the nurses from searching for information to answer the test and from copying from one another. The PEMAT was given to respondents at the end of the cloze test to evaluate their perceived understanding. This was done to prevent nurses from using the guide to respond to the cloze test.

### Data analysis

Text readability was assessed using a free online text readability checker consensus calculator (version 2.0). The two hundred and fifty (250) words from the PRC were selected and loaded into the consensus calculator for processing. Data were analyzed using the Flesch Reading Ease Score (FRES), Flesch-Kincaid Grade Level (FKGL), Gunning Fox Index (GFI), Coleman-Liau Readability Index (CLI), and the Simple Measure of Gobbledygook (SMOG). Scoring of items of the cloze test was done by calculating the percentage correct of the responses. A correct answer received one mark, and an incorrect answer received a 0 mark. Therefore, the percentage correct was determined by dividing the Total Correct Responses (TCR) by the Total Number of Test Items (TNTI) multiplied by hundred (percentage correct = [TCR/TNTI] x 100). The scores of the cloze test show the actual comprehension and scores from the PEMAT show the perceived understandability of the PRC. The nurses’ sociodemographic data was coded and entered into SPSS (version 26) together with the test items for processing and analysis. Descriptive statistics were used to present the sociodemographic characteristics, actual comprehension, and perceived understanding or otherwise of nurses. A paired t-test was done to assess the differences in the scores of the cloze test and the PEMAT. Multivariable regression was performed to establish the relationship between demographic information, comprehension, and knowledge. Data were presented using tables.

## Results

The results of the readability, comprehensibility, and their influence on knowledge of the PRC are presented. The readability indices of the PRC of Ghana are first presented followed by comprehensibility and lastly the influence of comprehension on knowledge.

### Readability test

Table 2 presents the results of the readability analysis. The FKRGL (13.36), SMOG (11.57), CL (14.2), and the average RGL indicator (14) show an individual will require at least 14 years of education to be able to read the PRC of Ghana. The GFI (15.40) and the FRES (34%) are concerned with the difficulty level of the passage. Both indices show that the PRC is difficult to read.

**Table 2:** Readability Indices of the PRC of Ghana (250-word passage)

Details	Readability Indices					
	Average RGL	FRE	FKRGL	GFI	SMOG	CL
PRC (250-word passage)	14	34%	13.63	15.40	11.57	14.2

Constructed by Researcher, August 2023

### Demographic characteristics of nurses

Table 3 presents the socio-demographic characteristics of the respondents. The study involved 205 nurses, the majority of whom were between the ages of 21–39 (91.7%). The mean age was 32 years. 74% (74.1%) were female and 25.9% were male. In terms of education, 61% had a diploma in nursing, and 27.3% had first degrees in nursing. 38% (38%) have practiced nursing between 3 and 4 years.

**Table 3:** Demographic Characteristics of Nurses (*n* = 205)

ITEM	FREQ (100%)	ITEM	FREQ (100%)
**Age**		**Number of Years of Work**	
18–20	-	1–2	70 (34.0%)
21–39	188 (91.7%)	3–4	78 (38.0%)
40–60	17 (9.3%)	5+	57 (28.0%)
60+	-		
Mean Age	32		
		**Rank**	
**Gender**		Senior Enrolled Nurse	22 (10.7%)
Male	53 (25.9%)	Enrolled Nurse	88 (42.9%)
Female	152 (74.1%)	Staff Nurse	67 (32.7%)
		Senior Staff Nurse	21 (10.2%)
		Principal Nursing Officer	3 (1.5%)
		Nursing Officer	4 (2.0%)
**Religion**			
Christianity	171 (83.4%)	**Place of Residence**	
Islam	34 (16.6%)	Agona Swedru	43 (21.0%)
Others	-	Breman Asikuman	48 (23.4%)
		Gomoa Akropong II	1 (0.5%)
		Kasoa	4 (2.0%)
		Winneba	109 (53.2%)
**Level of Education**			
Certificate	19 (9.3%)		
Diploma	125 (61.0%)		
Post Diploma	5 (2.4%)		
Degree	56 (27.3%)		

Source: Field data, August 2023

### Respondents’ comprehension of the PRC

The Cloze Test measured actual comprehensibility of the PRC and the PEMAT evaluated respondents perceived understanding of the 250-word passage used to construct the Cloze test. The results are presented in Table 4 and 5.

**Table 4:** Level of Comprehensibility of PRC Based on the Cloze Test (*n* = 205)

No. of Participants	Percentage	Correct Answer (%)	Level of Comprehension
179	87.3%	≥ 60 - ≤ 100	Independent Level– readers can cope with the language of the material
9	4.4%	≥ 40 - ˂ 60	Instructional level– readers unable to cope with the language of the materials (needing the support of an educator)
17	8.3%	0 ≥ - < 40	Frustration level– the language of the material is too difficult for readers to cope with or understand.
Mean marks in (%)		77.35%	

Constructed by Researcher, August 2023

**Table 5:** Nurses’ Perceived Understanding of PRC based on PEMAT

Question item	Frequency (*n* = 205)		
	Agree	Disagree	N/A
The material’s purpose is clearly defined, and the contents are summarized.	153 (74.6%)	53 (25.4%)	
The material does not contain any information that would turn your attention away from its purpose.	149 (72.7%)	56 (27.3%)	
The material uses common, everyday language. It does not include abbreviations or acronyms.	150 (73.2%)	55 (26.8%)	
Medical terms are explained and easily understood when introduced.	138 (67.3%)	67 (32.7%)	
The material may be personal or directed towards others.	118 (57.6%)	87 (42.4%)	
The material does not require the user to perform calculations.	136 (66.3%)	69 (33.7%)	
The material presents information in an orderly manner and makes sense.	154 (75.1%)	51 (24.9%)	
The material simplifies information for easy comprehension.	121 (59.0%)	39 (19.0%)	45 (22.0%)
The material’s sections have headers that provide users with a clear understanding of what to expect.	118 (57.5%)	43 (21.0%)	44 (21.5%)
The material summarizes the key points.	163 (79.5%)	24 (11.7%)	18 (8.8%)

Constructed by Researcher, August 2023

The Cloze Test revealed that a significant number (87.3%) of the respondents had scores between 60% and 100%, implying that they can cope with the language of the PRC of Ghana. Around 4.4% scored between 40% and 60%, which suggests they would need some level of explanation to be able to comprehend the PRC. Moreover, 8.3% scored below 40%, indicating reading frustration due to language difficulty. The PEMAT responses showed that a significant proportion (74.6%) of the respondents agreed that the material’s purpose was clear, it uses everyday language (73.2%), and presents medical terms in an easy-to-understand language (67.3%). More than half (59.0%) of the respondents said the material simplifies complex information. However, a few (22.0%) were neutral or uncertain about the charter’s clarity and disagreed with issues of clarity of its headers (21.0%). A paired sample T-test was performed to ascertain differences in scores between the Cloze Test and the PEMAT. The results show significant differences in the mean scores of the two tests 3.72; CI = 0.87–7.35 (t = 2.02; *P* = 0.04 < 0.05). (See Table 1 in the supplementary file for details).

### Knowledge of the PRC

Table 6 presents the nurses’ knowledge of the PRC. The results show none of them had extreme knowledge of the charter and 19.5% had no knowledge of it.

**Table 6:** Level of Knowledge of the PRC (*n* = 205)

No. of Participants	Percentage	Components Mentioned	Level of Knowledge
0	0.0%	5–6	Extreme Knowledge– able to recall 5–6 of the rights and responsibilities.
35	17.1%	3–4	Moderate Knowledge– able to recall 3–4 of the rights and responsibilities.
130	63.4%	1–2	Poor Knowledge– able to recall 1–2 of the rights and responsibilities.
40	19.5%	0	No Knowledge– unable to recall any of the rights and responsibilities.

Constructed by Researcher, August 2023

### Factors of the PRC knowledge

The regression model summary showed an adjusted r^2^ = 0.11, suggesting that the model explains around 11.0% of the variance in PRC knowledge. The ANOVA value [F (6,204) = 5.13, *P* = 0.01 r^2^ = 0.14] indicates that the model is statistically significant, explaining approximately 11% of the variance in knowledge scores. (See Table 2 in the supplementary file for the summary model of the regression analysis). As presented in Table 7, the regression coefficients show a significant positive effect of actual comprehension (β = 0.31; *p* = 0.01) on knowledge of the PRC. Respondents’ professional rank (β = 0.10 *p* = 0.20), Age (β = 0.003 *p* = 0.72), years of work (β = 0.08 *p* = 0.31), and rank (β = 0.10 *p* = 0.20) were immaterial in predicting knowledge of the PRC. While perceived understandability (β=-0.08; *p* = 0.25) and education (β=-0.07; *p* = 0.31) had no significant relationship with education, they were also negatively related to knowledge.

**Table 7:** Regression Analysis of Factors Influencing PRC Knowledge

	Standard Error	Standardized Beta	T	Sig.	VIF
Constant	11.24		0.39	0.70	
Age	4.59	0.03	0.36	0.72	1.21
Level of Education	1.27	-0.07	-0.91	0.36	1.19
Years of Work	1.64	0.08	1.02	0.31	1.24
Rank	1.41	0.10	1.23	0.20	1.47
Perceived Understandability	0.07	-0.08	-1.15	0.25	1.03
Actual Comprehension	0.06	0.31	4.60	0.01	1.07

Source Field data, August 2023. Significant at 5%

## Discussions

This study sought to assess nurses’ knowledge of the Patient Rights Charter of Ghana and associated literacy-related factors Literacy and knowledge of the PRC are essential inputs for centered care and patient experience. The charter is an embodiment of ethical, respectful, and nondiscriminatory healthcare delivery [23]. From the findings, the PRC has a higher RGL and may be difficult to read as shown by the FRES and the GFI. The results suggest that some nurses may require additional support to effectively read and comprehend its content. Nurses have a responsibility to advocate for the rights of their patients, and a comprehensive understanding of the PRC is essential for them to fulfill this duty effectively [24]. The finding also highlights the need for institutional-level management to consider simplifying the contents of the PRC. The summaries should use plain language and practical examples.

The findings of the actual comprehension and perceived understandability suggest that the PRC is generally comprehensible by nurses as most of them could cope with the language of the material without assistance. It is not surprising that most nurses were able to read and understand the PRC, which could be explained by the majority of nurses having a tertiary level of education and their awareness of the charter. Most nurses found it to be clear and concise. They appreciated the use of plain language and the avoidance of jargon and medical terminologies. Inferring from readability indices, one should have at least fourteen (14) years of education to be able to read and understand the PRC. However, a small percentage of the nurses found the language of the PRC difficult to understand, indicating that some nurses may require the help of an educator to be able to comprehend the charter. This aspect of the results could be attributed to the fact that a few of the respondents were certificate nurses. Quesenberry [25] highlights the importance of creating patient education materials in plain language to address low health literacy. It will help if educational programs on the PRC are provided at the facility level to effectively train and educate nurses about the PRC.

### Knowledge of the PRC

It is important for nurses to be knowledgeable about the PRC to contribute to quality healthcare delivery. In our study, nurses were asked to mention some rights and responsibilities of patients as enshrined in the PRC of Ghana. The authors expected respondents to mention at least three rights and three responsibilities. However, most nurses were able to mention between one (1) and two (2) rights or responsibilities, which means that the ability to read and understand may not translate to extensive knowledge of the charter. Cannon [26] argued that continuing education for nurses is key to addressing gaps in knowledge of patient education and literacy. A study by Souza [27] on nurses’ knowledge of patients’ rights in intensive care units found that nurses had a good overall understanding of patients’ rights, but there were some areas where they lacked knowledge. These findings highlight the need for comprehensive education on PRC among nurses. The core duty of a nurse is to draw up and implement nursing care plans for patients. Knowledge of patients’ rights is therefore critical for nurses to fulfill this role. The findings of this study indicate a need for significant improvement in nurses’ knowledge of the PRC. Prioritizing patient education and providing nurses with the necessary tools and resources will help them to effectively provide ethical care.

### Factors of the PRC knowledge

The study considered factors that could influence nurses’ PRC knowledge, including age, work experience, the rank of a nurse, perceived understandability, and actual comprehension. The professional rank of nurses, though not significant, suggests that nurses of higher rank may have higher PRC knowledge than those in the lower rank. This is possible because such nurses are more likely to be in decision-making positions such as heads of wards and other nursing units in district hospitals. Nurses in higher professional ranks are opportune to resolve patient-nurse misunderstandings. Thus, a higher-ranked nurse may attach much importance to nursing values. Although the literature is silent on the professional rank and knowledge of the PRC among nurses, the results corroborate findings on nursing performance [28]. In this particular study, a relationship was established between nurses in specialized hospitals and adherence to nursing care values [28]. Other studies on nurses’ performance have found no significant relationship between years of experience and the performance of nurses [29]. In spite of the immaterial relationship between rank and knowledge, Ericsson, Krampe, and Tesch-Römer [30] in their conceptual framework on deliberate practice and expert performance also suggested that deliberate practice often produces expert performance, which could be the case for nurses’ professional rank and knowledge of the PRC in this study.

While perceived understandability had a negative influence on knowledge and was insignificant, actual comprehension of the PRC was significantly influential in predicting PRC knowledge. The coefficients of perceived and actual understanding of the PRC tell us that we cannot use people’s subjective assessment of the linguistic characteristics of the PRC as a proxy measure for knowledge but rather, an objective assessment of its comprehensibility can help predict knowledge. A study by Adelberg & Razek [31] provides strong evidence that objective measures of comprehension are more reliable and valid than subjective measures. The mere display of the PRC at vantage points in hospitals may not lead to knowledge of it but motivating nurses to read with understanding can promote knowledge. The negative, though insignificant relationship between education and knowledge suggests that hospitals cannot depend only on the educational background of nurses to promote knowledge and awareness of the PRC of Ghana. While general literacy is important to knowledge, a deliberate effort at the institutional level to stimulate knowledge of the PRC among nurses and by extension, other clinicians is essential for ethical and centered care. The authors also observed that the coefficient of the constant term was not significant. This result suggests that the mean effect of all omitted variables may not be important, accentuating the value of comprehension to knowledge of the PRC among nurses.

## Conclusion

This study assessed nurses’ knowledge of the PRC of Ghana and associated literacy-related factors. To the best of the authors’ knowledge, this present study may be the first to delve into literacy-related challenges of the PRC of Ghana. The study revealed that the PRC has a higher readability level and may be difficult to read and comprehend by people with thirteen (13) or fewer years of education. The study also showed the PRC is generally comprehensible by nurses, but their knowledge of it is not as extensive as expected. Actual comprehension was the most significant predictor of PRC knowledge, while perceived understandability and education had a negative and insignificant influence on knowledge. Merely displaying the content of the PRC in hospitals will not result in knowledge and awareness. Rather, institutional-level initiatives, such as simplifying the content of the charter into easy-to-read educational material will help improve comprehension and knowledge. Other interventions such as sensitization and staff orientation on the PRC should be implemented to promote a broader understanding and knowledge. Education on the charter during customer care and other continuous improvement meetings at the facility level is recommended to stimulate knowledge and improve ethical and centered care.

### Electronic supplementary material

Below is the link to the electronic supplementary material.

Supplementary Material 1

Supplementary Material 2

Supplementary Material 3

Supplementary Material 4

## Data Availability

Future users, agencies, and researchers (within and outside Ghana) will be required to contact the authors to discuss the use of data. The research data will be made available on request based on evidence of ethical clearance from a recognized ethics review committee. All data requests should be sent to the corresponding author (John Foster Atta-Doku: johnkobbyjr@gmail.com).

## Abbreviations

PRC: Patient Rights Charter
SPSS: Statistical Package for Social Sciences
FRE: Flesch Reading Ease
FKGL: Flesch-Kincaid Grade Level
GFI: Gunning Fox Index
SMOG: Simple Measure of Gobbledygook
PEMAT: Patient Education Material Assessment Tool
GFR: Gunning Fox Readability
CLRI: Coleman-Liau Readability Index
