# Supplementary material for: Literacy-related factors and knowledge of patient rights charter: evidence from nurses in selected hospitals in Ghana
Source: BMC Nurs. 2024 Jan 22;23:60. doi: 10.1186/s12912-024-01739-w (PMC10801987; doi:10.1186/s12912-024-01739-w)
Supplement: Supplementary file 1 — Supplementary Material 1 [file 12912_2024_1739_MOESM1_ESM.docx]

**Supplementary Table 1: Differences between responses to the Cloze Test and the PEMAT (n=205)**

| ***Variable*** | ***Mean*** | ***Mean Dif.*** | ***Std. Dev.*** | ***t-statistics*** | ***P-value*** | ***95% Conf. Interval*** | |
| --- | --- | --- | --- | --- | --- | --- | --- |
| Cloze Test Score - | 77.35  73.63 | 3.72 | 26.36 | 2.02 | 0.04 0.87 | | 7.35 |
| PEMAT Test Score |  |  |  |  |  |  |  |

***Source: Field data, August 2023***

**Supplementary Table 2: Model Summary and ANOVA for Regression on Factors Affecting Knowledge**

|  | **R^2^** | **Adjusted R^2^** | **Standard Error** | **Sum of Squares** | **Dif** | **F** | **Sig.** |
| --- | --- | --- | --- | --- | --- | --- | --- |
| Knowledge |  |  |  |  | 6 | 5.13 | 0.00 |
|  | 0.14 | 0.11 | 16.48 | 8361.25 | 204 |  |  |

***Source: Field data, August 2023.***
