# Supplementary material for: Literacy-related factors and knowledge of patient rights charter: evidence from nurses in selected hospitals in Ghana
Source: BMC Nurs. 2024 Jan 22;23:60. doi: 10.1186/s12912-024-01739-w (PMC10801987; doi:10.1186/s12912-024-01739-w)
Supplement: Supplementary file 2 — Supplementary Material 2 [file 12912_2024_1739_MOESM2_ESM.docx]

**PASSAGE FOR READABILITY TEST**

The patient is entitled to personal safety and reasonable security of property within the confines of the institution. Hospital charges, modes of payments, and all forms of anticipated expenditures shall be explained to the patient prior to treatment. The patient is entitled to confidentiality of information obtained about him/her and such information shall not be disclosed to a third party without his/her consent, or the person entitled to act on his/her behalf except where such information is required by law or is in their public interest.

The patient has the right to a second medical opinion if he/she so deserves it. The right to quality basic health care irrespective of his/her geographical location. The right to consent or decline to participate in a proposed research study involving him or her after a full explanation has been given. The right to know the identity of his/her caregivers and other persons who may handle him/her including student trainees and ancillary. The right to know of alternative treatment and other healthcare providers within the service if they may contribute to improved outcomes.

The patient is entitled to all relevant information regarding policies and regulations of the healthcare facilities that he/she attends. Procedures for complaints, disputes, and conflict resolution shall be explained to patients or their accredited representatives. The patient has the right to privacy during the consultation, examination, and treatment. In cases it is necessary to use the patient or his/her case notes for teaching and conferences, the patient's consent must be sought.
