# Supplementary material for: Literacy-related factors and knowledge of patient rights charter: evidence from nurses in selected hospitals in Ghana
Source: BMC Nurs. 2024 Jan 22;23:60. doi: 10.1186/s12912-024-01739-w (PMC10801987; doi:10.1186/s12912-024-01739-w)
Supplement: Supplementary file 3 — Supplementary Material 3 [file 12912_2024_1739_MOESM3_ESM.docx]

**Greetings!**

This questionnaire is divided into three sections, each focusing on different aspects related to demographics, knowledge of the Patient Charter, and a comprehension assessment of patient rights using a Cloze Test. Your participation is highly valued, and your responses will remain confidential, and used solely for research purposes.

Thank you for dedicating your time to this survey. Your input is greatly appreciated.

**SECTION A: SOCIO-DEMOGRAPHIC CHARACTERISTICS OF NURSES.**

Please tick (√) and write as suitable in the boxes or blank spaces provided.

1. Age: 18-20 [ ] 21-39 [ ] 40-60 [ ] Above 60 [ ].
2. Gender: Male [ ] Female [ ]
3. Religion: Christianity [ ] Islam [ ] Others [ ]
4. Level of education: Certificate [ ] Diploma [ ] Post Diploma [ ] Degree [ ]
5. Number of years of work: 1-2 [ ] 3-4 [ ] 5+ [ ]
6. Rank: Senior Enrolled Nurse [ ] Enrolled Nurse [ ] Staff Nurse [ ]

Senior Staff Nurse [ ] Principal Nursing Officer [ ] Nursing Officer [ ]

1. Place of residence …………………………………………………………

**SECTION B: KNOWLEDGE OF THE PATIENT CHARTER**

Please respond to each question regarding your knowledge of the charter and its contents.

1. Have you heard of the Patient Charter?
2. Yes
3. No
4. Where did you hear of it?
5. Media
6. School
7. Health facility
8. Friends
9. Can you mention some responsibilities of patients stated in the Patients’ Charter?
10. Yes
11. No
12. If yes, mention

..............................................................................................................................................................................................................................................................................................................................................................................................................................

1. Can you mention some rights stated in the patients’ charter?
2. Yes
3. No
4. If yes, mention

..............................................................................................................................................................................................................................................................................................................................................................................................................................

**SECTION C: CLOZE TEST**

The Universal Declaration of Human Rights (1948) emphasizes all humans' fundamental dignity and equality. In recent decades, the concept of Patient Rights has been developed around the world based on this concept. The section aims to assess comprehension of the patient charter.

**Instruction**

There are 15 multiple-choice test questions grouped under themes. Answer each question by circling the alphabet of the correct option.

1. Patients have the right to know the ………………………. of his/her caregivers.
2. faith
3. identity
4. specification
5. uniqueness
6. The patient is entitled to all …………………... regarding policies and regulations of the healthcare facilities that he/she attends.
7. apposite themes
8. apposite tutorials
9. relevant application
10. relevant information
11. Hospital charges, modes of payments, and all forms of anticipated expenditures shall be explained to the patient …………………... treatment.
12. after
13. later in the
14. prior to
15. right after
16. Patients have the right to ………….…......other persons who may handle him/her including student trainees and ancillary.
17. endorse
18. know
19. instruct
20. inform
21. The patient is entitled to ………………... of information obtained about him/her.
22. clarity
23. confidentiality
24. the essentials
25. the relevance
26. Patient information shall not be ……………... to a third party without his/her consent, or the person entitled to act on his/her behalf.
27. concealed
28. confounded
29. disclosed
30. discluded
31. Patient information shall be …………... if required by law or is in their public interest.
32. transposed
33. exposed
34. expended
35. transcended
36. Patient has the right to ……………. during the consultation, examination, and treatment.
37. prove himself/herself.
38. enhance himself/herself.
39. Privacy.
40. be careful.
41. In the case of using the patient or their information for teaching and conferences, the patient's consent must be …………...
42. checked
43. questioned
44. sought
45. weighed
46. The patient is entitled to personal ………………. and reasonable security of property within the confines of the institution.
47. information
48. encounter
49. checks
50. safety
51. Patients have the right to quality …………… care irrespective of his/her geographical location.
52. basic
53. basic health
54. basic financing
55. basic health strategy
56. Patients who decide to........................ a research project are entitled to the most effective care available.
57. take control in
58. incur the cost of
59. participate in
60. sponsor
61. Patients who decide to ............................ a research project are entitled to the most effective care available.
62. enforce
63. conclude on
64. withdraw from
65. withhold
66. A patient is...................... full information on his or her condition and management.
67. charged with
68. responsible for
69. entitled to
70. to accept
71. A patient is entitled to know all possible risks involved in .........................except during an emergency.
72. transfer
73. treatment
74. financing
75. supplies
