# Supplementary material for: Literacy-related factors and knowledge of patient rights charter: evidence from nurses in selected hospitals in Ghana
Source: BMC Nurs. 2024 Jan 22;23:60. doi: 10.1186/s12912-024-01739-w (PMC10801987; doi:10.1186/s12912-024-01739-w)
Supplement: Supplementary file 4 — Supplementary Material 4 [file 12912_2024_1739_MOESM4_ESM.docx]

**The Patient Education Material Assessment Tool (PEMAT)**

**Instruction**

There are 10 sets of questions in tabular form. Enter 0 if you “Disagree” or 1 if you “Agree.” Some but not all items will also have a “Not Applicable” answer option.

| **No.** | **Item** | **Response Options** | **Rating** |
| --- | --- | --- | --- |
| 1 | The purpose of the material is clearly stated. It uses a title that summarizes the content. | Disagree=0, Agree=1 |  |
| 2 | The material does not contain any information that would turn your attention away from its purpose. | Disagree=0, Agree=1 |  |
| 3 | The material uses common, everyday language. It does not include abbreviations or acronyms. | Disagree=0, Agree=1 |  |
| 4 | The only purpose of using medical terms is to introduce the audience to them. The medical terms are simple to understand when used. | Disagree=0, Agree=1 |  |
| 5 | Basically, the material makes what you read more personal. When this is the case, tick agree. But in some few cases, it may be directed toward other people. When this is the case, still agree. | Disagree=0, Agree=1 |  |
| 6 | The material does not expect the user to perform calculations. A calculation is when the material asks the user to add, subtract, multiply, divide, or perform any other mathematical operation. | Disagree=0, Agree=1 |  |
| 7 | The material breaks information down for easy understanding. Choose N/A if the material meets the definition of a very short material. | Disagree=0, Agree=1  Very short material=N/A |  |
| 8 | The material’s sections have informative headers. Headings are specific and the user knows what to expect in that section. Choose N/A if the material meets the definition of a very short material. | Disagree=0, Agree=1  Very short material=N/A |  |
| 9 | The material presents information in an orderly manner and makes sense. | Disagree=0, Agree=1 |  |
| 10 | The material summarizes the key points. Choose N/A if the material does not meet its definition. | Disagree=0, Agree=1  Very short material=N/A |  |

**250-words Passage for PEMAT**

The patient is entitled to personal safety and reasonable security of property within the confines of the institution. Hospital charges, modes of payments, and all forms of anticipated expenditures shall be explained to the patient prior to treatment. The patient is entitled to confidentiality of information obtained about him/her and such information shall not be disclosed to a third party without his/her consent, or the person entitled to act on his/her behalf except where such information is required by law or is in their public interest.

The patient has the right to a second medical opinion if he/she so deserves it. The right to quality basic health care irrespective of his/her geographical location. The right to consent or decline to participate in a proposed research study involving him or her after a full explanation has been given. The right to know the identity of his/her caregivers and other persons who may handle him/her including student trainees and ancillary. The right to know of alternative treatment and other healthcare providers within the service if they may contribute to improved outcomes.

The patient is entitled to all relevant information regarding policies and regulations of the healthcare facilities that he/she attends. Procedures for complaints, disputes, and conflict resolution shall be explained to patients or their accredited representatives. The patient has the right to privacy during the consultation, examination, and treatment. In cases it is necessary to use the patient or his/her case notes for teaching and conferences, the patient's consent must be sought.
